# Development and Validation of a 15-gene Expression Signature with Superior Prognostic Ability in Stage II Colorectal Cancer

**DOI:** 10.1158/2767-9764.CRC-22-0489

**Published:** 2023-08-30

**Authors:** Matjaz Rokavec, Elif Özcan, Jens Neumann, Heiko Hermeking

**Affiliations:** 1 Experimental and Molecular Pathology, Institute of Pathology, Faculty of Medicine, Ludwig-Maximilians-Universität München, Munich, Germany.; 2 Institute of Pathology, Faculty of Medicine, Ludwig-Maximilians-Universität München, Munich, Germany.; 3 German Cancer Consortium (DKTK), Partner site Munich, Munich, Germany.; 4 German Cancer Research Center (DKFZ), Heidelberg, Germany.

## Abstract

Currently, there is no consensus about the use of adjuvant chemotherapy for patients with stage II colorectal cancer. Here, we aimed to identify and validate a prognostic mRNA expression signature for the stratification of patients with stage II colorectal cancer according to their risk for relapse. First, publicly available mRNA expression profiling datasets from 792 primary, stage II colorectal cancers from six different training cohorts were analyzed to identify genes that are consistently associated with patient relapse-free survival (RFS). Second, the identified gene expression signature was experimentally validated using NanoString technology and computationally refined on primary colorectal cancer samples from 205 patients with stage II colorectal cancer. Third, the refined signature was validated in two independent publicly available cohorts of 166 patients with stage II colorectal cancer. Bioinformatics analysis of training cohorts identified a 61-gene signature that was highly significantly associated with RFS (HR = 37.08, *P* = 2.68*10^−106^, sensitivity = 89.29%, specificity = 89.61%, and AUC = 0.937). The experimental validation and refinement revealed a 15-gene signature that robustly predicted relapse in three independent cohorts: an in-house cohort (HR = 20.4, *P* = 8.73*10^−23^, sensitivity = 90.32%, specificity = 80.99%, AUC = 0.812), GSE161158 (HR = 5.81, *P* = 3.57*10^−4^, sensitivity = 64.29%, specificity = 81.67%, AUC = 0.796), and GSE26906 (HR = 7.698, *P* = 7.26*10^−8^, sensitivity = 61.54%, specificity = 78.33%, AUC = 0.752). In the pooled training cohort, the 15-gene signature (HR = 4.72, *P* = 7.76*10^−25^, sensitivity = 75%, specificity = 67.44%, AUC = 0.784) was superior to the Oncotype DX colon 7-gene signature (HR = 2.698, *P* = 6.3*10^−8^, sensitivity = 62.16%, specificity = 55.5%, AUC = 0.633). We report the identification and validation of a novel mRNA expression signature for robust prognostication and stratification of patients with stage II colorectal cancer, with superior performance in the analyzed validation cohorts when compared with clinicopathologic biomarkers and signatures currently used for stage II colorectal cancer prognostication.

**Significance:**

We identified and validated a 15-gene expression signature for robust prognostication and stratification of patients with stage II colorectal cancer, with superior performance when compared with currently used biomarkers. Therefore, the 15-gene expression signature has the potential to improve the prognostication and treatment decisions for patients with stage II colorectal cancer.

## Introduction

Colorectal cancer is the second leading cause of cancer mortality and caused almost 900,000 deaths globally in the year 2018 (1). The prognosis of patients with colorectal cancer is mainly determined by staging, which also helps to make treatment decisions. There is no consensus about the use of adjuvant chemotherapy for patients with stage II colorectal cancer. The survival rate of patients with stage II colorectal cancer is relatively high, but approximately 20% of patients relapse within 5 years (2). Studies have found only 2%–5% improvement in survival with the addition of chemotherapy for stage II patients (3–5). This has to be weighed against the side effects of the chemotherapy treatment (6). Stratification of patients with stage II colorectal cancer according to their risk of relapse may allow the identification of patients who should benefit most from adjuvant chemotherapy (patients with high risk of relapse) and would limit overtreatment of patients for whom the toxicities of adjuvant chemotherapy outweigh the relatively low benefits (low-risk patients). The identification of high-risk stage II patients is challenging due to the lack of specific and sensitive biomarkers (7). Pathologic tumor invasion stage (pT) is significantly associated with survival of patients with stage II colorectal cancer (3, 8). However, invasion of the serosa or adjacent organs may be difficult to recognize and differentiate from non-neoplastic conditions, such as inflammation, presenting a challenge in staging (9). Besides clinicopathologic factors, gene expression signatures have been developed to stratify patients with stage II colorectal cancer (10). The most widely used and validated signature is the Oncotype DX colon 12-gene signature (Exact Sciences) consisting of the expression levels of seven recurrence genes and five reference genes (11). However, the clinical performance of currently available gene expression signatures has precluded their use as prognostic tests in the clinical setting (2, 6). By analyzing multiple training and validation cohorts of patients with stage II colorectal cancer, we developed and validated a 15-gene signature that allows the stratification of patients according to their risk of relapse with higher sensitivity and specificity than the currently used biomarkers. In the future, this 15-gene signature may guide physicians in determining the prognosis and making treatment decisions for patients with stage II colorectal cancer after surgical removal of the primary tumor.

## Materials and Methods

### Study Design and Patient Cohorts

**FIGURE 1:**
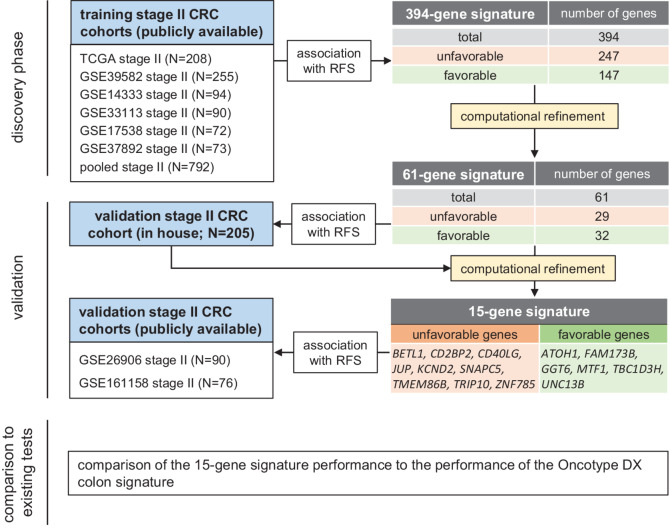
Study design and workflow scheme. The analysis of six publicly available colorectal cancer patient training cohorts identified 394 genes that were significantly associated with RFS in the majority of cohorts. The 394-gene signature was computationally refined to a 61-gene signature, which showed the highest prognostic significance in the training cohorts. The 61-gene signature was validated in the in-house validation cohort and further refined to a 15-gene signature, which was then validated in additional publicly available cohorts.

This study employed a three-phase design: initial discovery phase in training patient cohorts, first validation phase in the in-house validation cohort, and a second validation phase in additional validation cohorts (Fig. 1). In the discovery phase, transcriptomic and clinico-pathologic data from tumors of six publicly available colorectal cancer patient cohorts was analyzed (training cohorts: TCGA COAD+READ, GSE39582, GSE14333, GSE33113, GSE17538, GSE37892). Only samples from stage II patients from these cohorts were included in the analyses. The cohorts were analyzed individually or combined as a pooled training cohort. For the first validation cohort (in-house validation cohort), formalin-fixed and paraffin-embedded (FFPE) specimen of 210 patients with Unio Internationale Contra Cancrum (UICC) stage II colorectal cancer who underwent surgical resection at the Department of General, Visceral and Transplant Surgery, LMU Munich (Munich, Germany) between 1994 and 2007 were selected from the archives of the Institute of Pathology, Medical Faculty, LMU Munich ((Munich, Germany; Table 1). The corresponding clinico-pathologic data were obtained from the database of the Munich Cancer Registry. Specimens were anonymized, and the study was performed according to the recommendations of the local ethics committee of the Medical Faculty of the LMU Munich. In addition, transcriptomic and clinico-pathologic data from tumors of two publicly available validation cohorts of 166 patients with stage II colorectal cancer was analyzed (GSE26906 and GSE161158). Only samples with complete expression and clinical data were included in the analyses.

**TABLE 1:** Patient/tumor characteristic and their associations with the expression of the 15-gene signature in the in-house stage II colorectal cancer validation cohort

Characteristic	15-gene signature low	15-gene signature high	*P*
Total (*N* = 205)	122	83	
Age, mean	69.73	68.42	0.4482^a^
Gender			
Male	65	45	0.9999^b^
Female	56	38	
Grade			
1	3	0	0.114^c^
2	60	55	
3	58	28	
pT			
3	106	58	0.0022^b^
4	15	25	
KRAS			
wt	76	50	0.7699^b^
mut	45	33	

NOTE: *P* values were calculated with ^a^*t* test, ^b^Fisher exact test, or ^c^*χ*^2^ test for trend.

### Patient Consent for Publication

This study was carried out according to the recommendations of the ethics committee of the Medical Faculty of the Ludwig-Maximilians-University Munich, Munich, Germany. The current study has been performed in a retrospective manner in a cohort of patients diagnosed and treated according to national guidelines. In addition, the dataset as well as the specimens were irreversibly anonymized prior to inclusion in the study. Hence, under the circumstances aforementioned, neither a written consent nor a project-specific approval by the ethic committee was necessary. The study was performed according to the standards set in the declaration of Helsinki 1975. All researchers were blinded from patient data during experimental analysis.

### Ethics Approval

This study used tumor tissue that had initially been collected for histopathologic diagnostics. At the time, the tissue was examined for the current study all diagnostic procedures had already been fully completed and the tissue used was thus classified as leftover material. All patient data were fully anonymized. As per declaration of our ethics committee [Ludwig-Maximilians-University Medical School (Munich, Germany)] no written informed consent of the participants is needed given the circumstances described above.

### mRNA Expression Analysis

Transcriptomic data from publicly available training and validation cohorts were generated with microarrays, except for The Cancer Genome Atlas (TCGA), where mRNA expression was analyzed by RNA sequencing (RNA-Seq). For microarray and RNA-Seq datasets, normalized intensity signal data and RNA-Seq by expectation-maximization (RSEM) data were used, respectively. For genes targeted by multiple microarray probes, the sum expression of all probes for each gene was used. To generate the pooled training cohort dataset, transcriptomic data of individual training cohorts were first z-score normalized for each mRNA across every tumor and then merged (12, 13).

For the in-house validation cohort, tumor tissue was available as FFPE blocks. The FFPE blocks were cut to sections. One section was stained with hematoxylin and eosin and the tumor area was marked by a pathologist. RNA was isolated from the marked area by using the QIAGEN Rneasy FFPE kit according to manufacturer's instructions. The expression of 61 test mRNAs and six housekeeping mRNAs (*AKAP1, DNAJC14, SF3A1, TBC1D10B, TKL2,* and *TMUB2*) was analyzed with a NanoString nCounter MAX/Flex system. The housekeeping genes were selected on the basis of the study from Xu and colleagues, who determined the most suitable reference genes for colorectal cancer with the lowest variation in expression between tumors (14). The reporter codeset and capture probeset were mixed with 250 ng RNA. Capture codeset was added and incubated at 65°C for 18 hours. The hybridized samples were analyzed with a NanoString nCounter MAX/Flex system according to manufacturer's instructions. The sequences of NanoString probes are available on request. The person who performed the expression analyses was blinded to clinical data. The raw expression counts were normalized in two steps. The first step was positive control normalization in which six diluted positive controls were used for normalization between samples and lanes to remove variations arising from handling differences, hybridization, magnetic bead purification, slide-binding, and imaging. The second step was the normalization to the expression of the housekeeping genes. For quality control, the normalized data were analyzed using principle component analysis and relative log expression analysis. Five samples displayed dispersed mRNA expression counts and were therefore removed from further analyses.

### Statistical Analysis

Kaplan–Meier (KM) analysis was performed to determine survival outcomes [relapse-free survival (RFS) and overall survival (OS)] and the statistical significance was evaluated by the log-rank test. For binary classification of cases (high/low expression), the Survminer R package (https://cran.r-project.org/web/packages/survminer/index.html) was used to determine optimal cut-off values. Multivariate analysis was performed by the Cox proportional hazards regression model using the “survival” R package (https://cran.r-project.org/web/packages/survival/index.html). ROC curves, AUC, sensitivity, and specificity values were calculated using the GraphPad Prism 8.0 software. The association of signature-expression-recurrence-scores with the risk of recurrence was calculated using a generalized linear model using the pec R package (https://cran.r-project.org/web/packages/pec/index.html).

### Calculation of Recurrence Scores for Gene Signatures

To generate the recurrence score values for signatures in each tumor sample, we established a scoring system. First, the optimal cut-off values that classify cases to high/low expression according to the RFS outcome were determined for each gene in every cohort. Next, we used a scoring system that assigns 0 or 1 to every gene relative to the cut-off value. In case of unfavorable genes that are related to poor survival, a score of 1 was assigned to the samples in which mRNA levels were higher than the cut-off value and 0 for samples in which mRNA levels were below the cut-off value. For the favorable genes that are related to good survival, a score 1 was assigned to samples with mRNA levels below the cut-off value and 0 to samples with mRNA levels above the cut-off value. To generate recurrence scores for signatures, recurrence score values for all genes of the signature were summed up across every tumor sample. A high recurrence score indicates that the tumor has high expression of unfavorable genes and low expression of favorable genes. The Oncotype DX recurrence score (RS_U_) was additionally calculated on the basis of the formula provided by Gray and colleagues [RS_U_ = 0.15 × (*BGN + FAP + INHBA*)/3 − 0.3 × (*MYBL2 + KI67 + MYC*)/3 + 0.15 × *GADD45B*] (15).

### Computational Refinement of Gene Signatures

To identify an optimal signature that shows the most significant association with RFS, computational refinement was applied. First, the significance (log-rank *P* value) of the association between the recurrence score of every combination of two genes from a signature and RFS was calculated. Next, 2,000 most significant 2-gene combinations were chosen and one more gene from the remaining signature genes was added to obtain 3-gene combinations, for which the association between RFS and the recurrence score was calculated. This approach was continued until the number of combined genes reached the number of genes in the signature. Thereby, the signature with the most significant log-rank *P* value was obtained for every *n*-gene combination.

### Data Availability

The expression profile data analyzed in this study were obtained from TCGA data portal (https://portal.gdc.cancer.gov/) and the Gene Expression Omnibus (https://www.ncbi.nlm.nih.gov/geo/) at GSE39582, GSE14333, GSE33113, GSE17538, GSE37892, GSE2906, and GSE161158. The code for the computational refinement of gene signatures was deployed at https://github.com/MatjazRokavec/Stage2-CRC.

## Results

### Identification of a Prognostic Gene Expression Signature for Stage II Colorectal Cancer

First, we aimed to identify genes that are significantly and consistently associated with RFS in multiple stage II colorectal cancer patient cohorts. Therefore, we analyzed transcriptomic and clinical data from stage II tumors of six publicly available colorectal cancer training cohorts (TCGA COAD+READ, GSE39582, GSE14333, GSE33113, GSE17538, GSE37892). By performing KM analysis of all genes, we identified 394 genes whose expression was significantly associated with RFS in at least four from six patient cohorts (Supplementary Table S1). A total of 247 genes showed an unfavorable association, which means that high expression of these genes was associated with poor survival and 147 genes showed a favorable association, which means that high expression was associated with good prognosis. Unfavorable genes showed the highest enrichment for the epithelial–mesenchymal transition (EMT) and focal adhesion MSigDB gene sets (Supplementary Fig. S1A and S1B). Favorable genes did not show any significant enrichments. We analyzed whether any of the unfavorable genes might represent potential therapeutic targets for colorectal cancer treatment. Phosphoglycerate kinase 1 (PGK1), which was consistently associated with poor prognosis in training cohorts (Supplementary Fig. S1C) also displayed a strong dependency in colorectal cancer cell lines (Supplementary Fig. S1D; data from the cancer dependency map project). Moreover, *PGK1* mRNA expression was consistently significantly elevated in colorectal cancer tumors compared with matched normal colonic tissue in 13 from 14 cohorts (Supplementary Fig. S1E). Elevated expression of PGK1 has been already associated with chemotherapy and radiotherapy resistance and poor prognosis in multiple types of cancer (16). Therefore, PGK1 might represent a potential therapeutic target for colorectal cancer.

**FIGURE 2:**
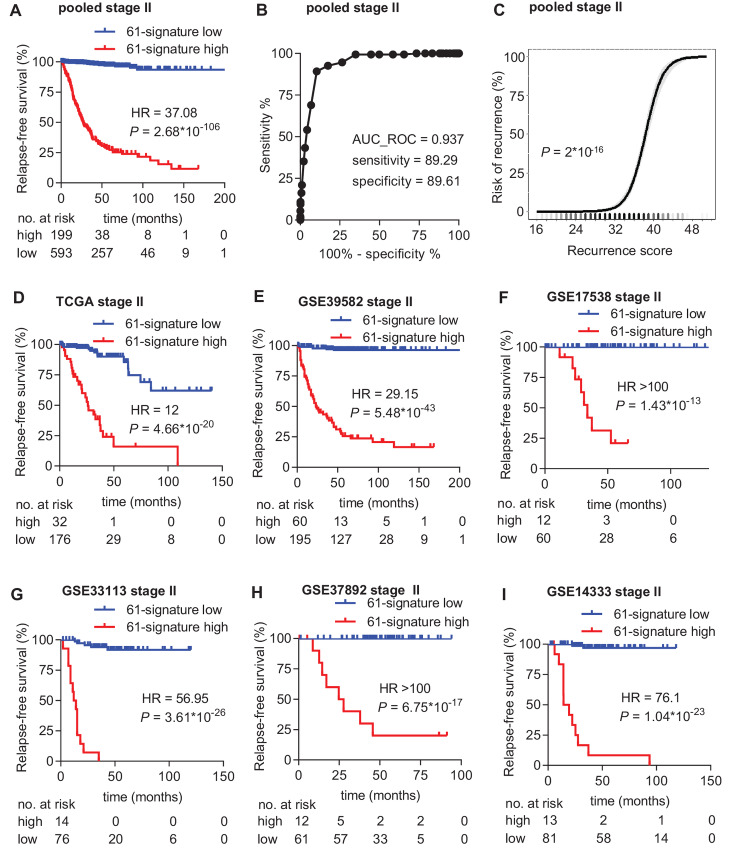
Performance of the 61-gene signature in predicting RFS in training cohorts. **A,** KM analysis of RFS according to the 61-gene signature classifier in the pooled training dataset. **B,** ROC graph showing the sensitivity, specificity and AUC value of the 61-gene signature to predict relapse in the pooled training dataset. The red dot indicates the recurrence score threshold that was used to determine sensitivity and specificity. **C,** Risk of recurrence as a continuous function of the 61-gene signature recurrence score within 10 years after surgery in the pooled training dataset. The shaded area around the curve represents the lower and upper 95% confidence interval (CI). **D**–**I,** KM analysis of RFS according to the 61-gene signature classifier in the indicated training datasets.

Next, we merged the transcriptomic and clinical data of the six training cohorts into one pooled training cohort. To identify an optimal signature that shows the most significant association with RFS, we applied computational refinement of the 394 genes in the pooled training cohort as described in Materials and Methods. Thereby, we identified a subset of 61 genes (32 favorable and 29 unfavorable genes; Supplementary Table S2), which showed a highly significant association with RFS in the pooled training cohort (HR = 37.08; log-rank *P* value = 2.68*10^−106^; Fig. 2A). ROC analysis showed that the 61-gene signature had a sensitivity of 89.29% and a specificity of 89.61% with an AUC value of 0.937 to predict relapse in the pooled training cohort (Fig. 2B). Next, a recurrence score was determined for each tumor, based on the expression of 61 genes as described in Materials and Methods. In the pooled training cohort, the risk of recurrence at 10 years increased continuously and significantly (*P* = 2*10^−16^) with increasing recurrence score (Fig. 2C). Importantly, the 61-gene signature also showed highly significant association with RFS in each of the individual six training cohorts (Fig. 2D–I; Supplementary Table S3). These results demonstrate the robustness of the 61-gene signature for the prediction of relapse in patients with stage II colorectal cancer.

### Validation and Further Development of the Prognostic Gene Expression Signature for Stage II Colorectal Cancer

**FIGURE 3:**
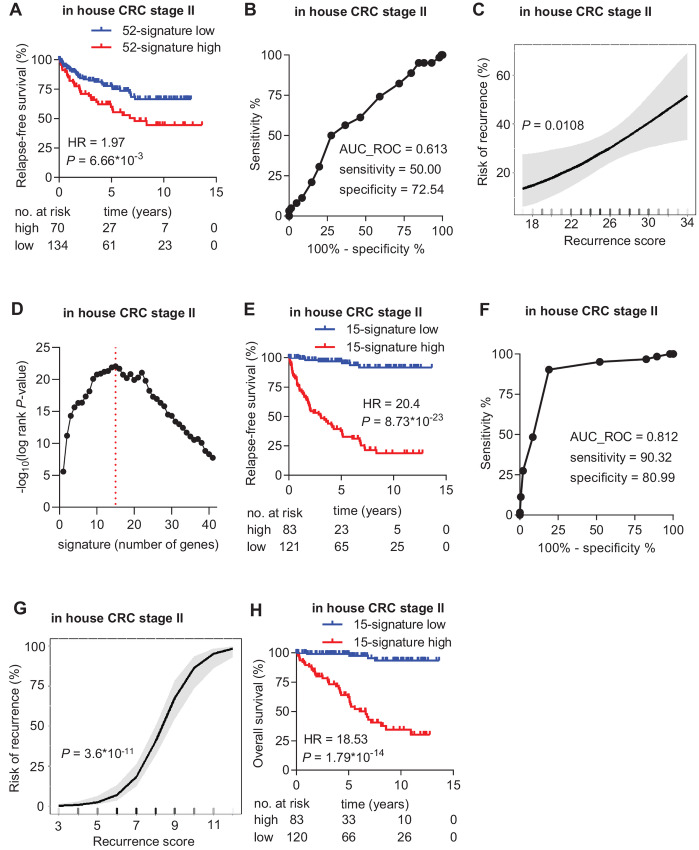
Validation and refinement of the prognostic gene signature in the in-house cohort. **A,** KM analysis of RFS according to the 52-gene signature classifier (derived from the 61-gene signature) in the in-house cohort. **B,** ROC graph showing the sensitivity, specificity, and AUC value of the 52-gene signature to predict relapse in the in-house cohort. The red dot indicates the recurrence score threshold that was used to determine sensitivity and specificity. **C,** Risk of recurrence as a continuous function of the 52-gene signature recurrence score within 10 years after surgery in-house cohort. The shaded area around the curve represents the lower and upper 95% CI. **D,** Significance [−log_10_(log-rank *P* value)] of the most significant signature (*y*-axis) for every *n*-gene combination (*x*-axis). **E,** KM analysis of RFS according to the 15-gene signature classifier in the in-house cohort. **F,** ROC graph showing the sensitivity, specificity and AUC value of the 15-gene signature to predict relapse in the in-house cohort. The red dot indicates the recurrence score threshold that was used to determine sensitivity and specificity. **G,** Risk of recurrence as a continuous function of the 15-gene signature recurrence score within 10 years after surgery in the in-house cohort. The shaded area around the curve represents the lower and upper 95% CI. **H,** KM analysis of OS according to the 15-gene signature classifier in the in-house cohort.

Next, we utilized an in-house validation cohort of 205 tumors from patients with stage II colorectal cancer to validate the identified prognostic signature (Table 1). By using the NanoString technology, the mRNA expression of 61 genes was analyzed. Nine genes showed a very low average expression (<10 counts/sample) and were therefore excluded from further analysis. The excluded genes were *CCDC96, MRPL22, SPATS1, CFC1B, GRIN3B, MYLK2, RTP3, SLC6A13*, and *TRDN*. All of these genes, except *MRPL22*, also showed low expression levels in public cohorts. Furthermore, based on the data from the Protein Atlas, the protein levels of these genes (except MRPL22) are low in colorectal cancer cells. Finally, a Pubmed search of these genes did not reveal any prominent role of these genes in cancer. Therefore, we believe that these genes do not play an important role in colorectal cancer. The signature of the remaining 52 genes was significantly associated with RFS in the in-house validation cohort (HR = 1.97; *P* = 6.66*10^−3^; Fig. 3A). The 52-gene signature showed a sensitivity of 50% and a specificity of 72.54% with an AUC value of 0.613 to predict relapse (Fig. 3B). The risk of recurrence at 10 years increased continuously and significantly (*P* = 0.0108) as the recurrence score increased (Fig. 3C). Although the 52-gene signature, which was derived from the 61-gene signature, was significantly associated with RFS in the validation cohort, the significance, sensitivity, and specificity was lower than in the training cohorts. To optimize the signature with respect to the prediction of relapse, we performed a computational refinement of the 52-gene signature as described in Materials and Methods. The significance of the association with RFS increased with increasing number of genes per signature up to 15 genes, but then decreased when more genes were added to the signature (Fig. 3D). Therefore, a subset of 15 genes (nine unfavorable genes: *BET1L*, *CD2BP2*, *CD40LG*, *JUP*, *KCND2*, *SNAPC5*, *TMEM86B*, *TRIP10*, *ZNF785* and six favorable genes: *ATOH1*, *FAM173B*, *GGT6*, *MTF1*, *TBC1D3H*, *UNC13B*), showed the most significant association with RFS in the in-house validation cohort (HR = 20.4; log-rank *P* value = 8.73*10^−23^; Fig. 3E). The 15-gene signature had a sensitivity of 90.32% and a specificity of 80.99% with an AUC value of 0.812 to predict relapse in the in-house validation cohort (Fig. 3F). The risk of recurrence at 10 years increased continuously and significantly (*P* = 3.6*10^−11^) with increasing recurrence score (Fig. 3G). Therefore, the refined 15-gene signature represents a better prognostic biomarker than the initial 61-gene signature in the in-house cohort. The 15-gene signature was also significantly associated with OS in the in-house validation cohort (HR = 18.53; log-rank *P* value = 1.79*10^−14^; Fig. 3H). The expression of the 15-gene signature was significantly associated with pT (Table 1). However, multivariate Cox regression analysis after adjustment for clinicopathologic variables pT and grade revealed that the 15-gene signature represents an independent prognostic factor for RFS and OS (Table 2). Moreover, the 15-gene signature showed a markedly stronger association with RFS and OS than the currently used clinicopathologic biomarker pT (Table 2).

**TABLE 2:** Multivariate Cox proportional hazards models for estimating the contribution of indicated variables to predict recurrence in indicated patient cohorts

Variable	HR RFS (95% CI)	*P*	HR OS (95% CI)	*P*
In-house validation cohort				
pT (4 vs. 3)	2.39 (1.39–4.12)	0.00171	2.08 (1.08–4.02)	0.0289
Grade (3 vs. 1+2)	0.69 (0.39–1.22)	0.204	0.67 (0.34–1.33)	0.2569
15-gene signature (high vs. low)	19.45 (8.29–45.60)	8.94*10^−12^	17.01 (6.02–48.05)	8.88*10^−8^
TCGA stage 2				
pT (4 vs. 3)	1.84 (0.51–6.59)	0.3483	2.33 (0.68–7.94)	0.1773
MSS vs. MSI	1.03 (0.43–2.46)	0.9552	0.68 (0.32–1.41)	0.2967
15-gene signature (high vs. low)	8.53 (3.92–18.57)	6.63*10^−8^	21.23 (2.94–155.9)	0.0027
GSE39582 stage 2				
pT (4 vs. 3)	2.45 (1.29–4.66)	0.00625	1.23 (0.64–2.37)	0.5402
MSS vs. MSI	1.59 (0.64–3.98)	0.3167	0.93 (0.45–1.93)	0.8475
15-gene signature (high vs. low)	6.57 (3.52–12.25)	3.39*10^−9^	2.72 (1.40–5.29)	0.0031
GSE33113 stage 2				
pT (4 vs. 3)	1.52 (0.33–7.10)	0.5931	NA	NA
Grade (3 vs. 1+2)	3.69 (1.23–11.09)	0.0198	NA	NA
MSS vs. MSI	1.24 (0.38–3.98)	0.722	NA	NA
15-gene signature (high vs. low)	36.79 (9.38–144.28)	2.32*10^−7^	NA	NA

Abbreviation: CI, confidence interval.

Other patient cohorts were not analyzed, because clinicopathologic data beside survival were not available.

**FIGURE 4:**
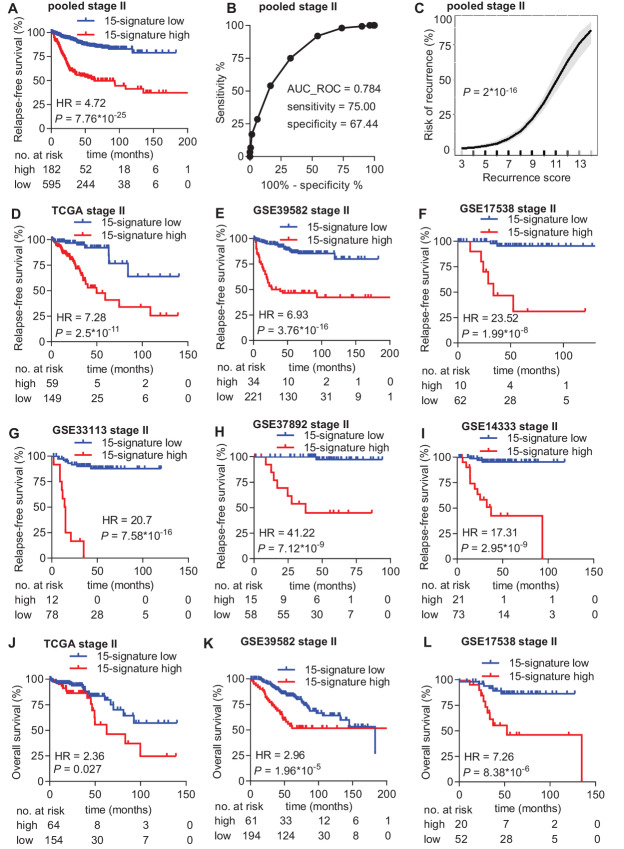
Performance of the 15-gene signature in predicting RFS in training cohorts. **A,** KM analysis of RFS according to the 15-gene signature classifier in the pooled training dataset. **B,** ROC graph showing the sensitivity, specificity, and AUC value of the 15-gene signature to predict relapse in the pooled training dataset. The red dot indicates the recurrence score threshold that was used to determine sensitivity and specificity. **C,** Risk of recurrence as a continuous function of the 15-gene signature recurrence score within 10 years after surgery in the pooled training dataset. The shaded area around the curve represents the lower and upper 95% CI. **D**–**I,** KM analysis of RFS according to the 15-gene signature classifier in the indicated training datasets. **J**–**L,** KM analysis of OS according to the 15-gene signature classifier in the indicated training datasets.

**FIGURE 5:**
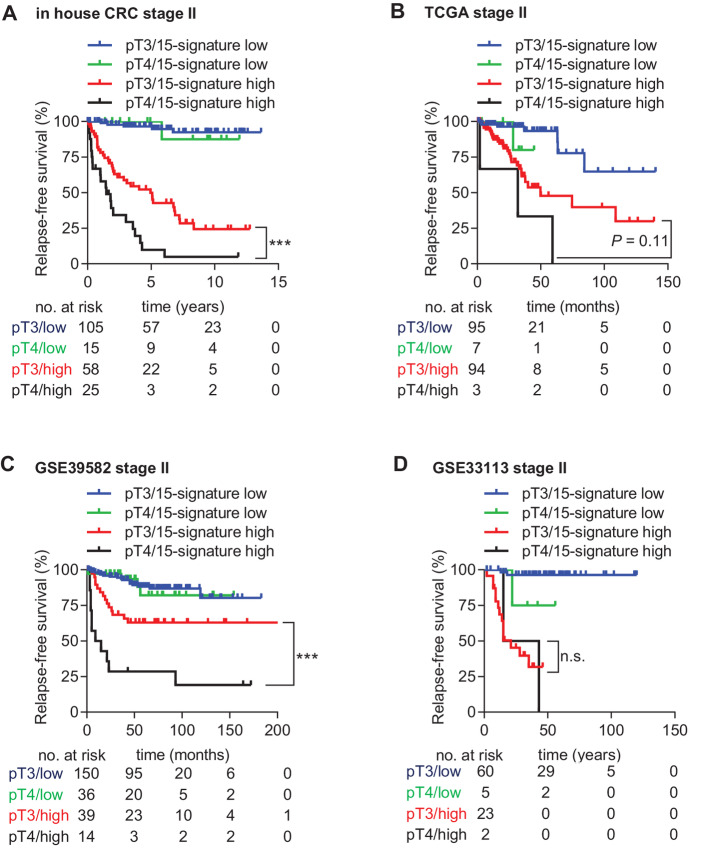
Performance of the combined 15-gene signature/pT classifier in predicting RFS. **A**–**D,** KM analysis of RFS according to the 15-gene signature score combined with the pT classifier in the indicated stage II colorectal cancer patient cohorts.

Next, we analyzed the 15-gene signature in the six publicly available colorectal cancer training cohorts that were initially used to identify RFS-associated genes. The 15-gene signature was significantly associated with RFS in the pooled training cohort, consisting of all six training cohorts (HR = 4.72; *P* = 7.76*10^−25^; Fig. 4A). The signature showed a sensitivity of 75% and a specificity of 67.44% with an AUC value of 0.784 to predict relapse (Fig. 4B) and the risk of recurrence at 10 years increased continuously and significantly (*P* = 2*10^−16^) with increasing recurrence score (Fig. 4C). Notably, the 15-gene signature was significantly associated with RFS in each of the six training cohorts (Fig. 4D–I; Supplementary Table S3). Moreover, the 15-gene signature was significantly associated with OS in three training cohorts for which the OS data were available (Fig. 4J–L). Finally, multivariate Cox regression analysis after adjustment for clinicopathologic variables pT and microsatellite instability (MSI) status showed that the 15-gene signature represents an independent and most significant prognostic factor for RFS and OS in three cohorts for which clinicopathologic data other than survival was available (Table 2). Yet, the inclusion of pT improved the accuracy of relapse prediction for patients with high 15-gene score. Among patients with high 15-gene score, pT4 patients had significantly worse outcome than pT3 patients in the in-house and the GSE39582 cohort (Fig. 5A–D).

**FIGURE 6:**
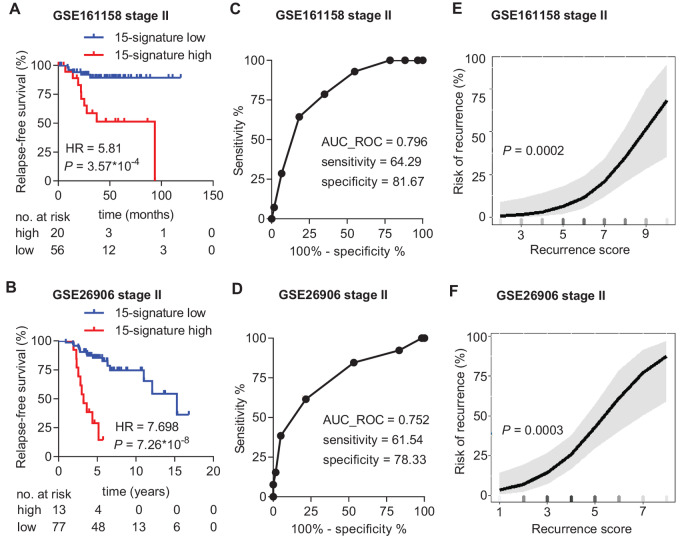
Validation of the 15-gene signature in predicting RFS in additional colorectal cancer cohorts. **A,** KM analysis of RFS according to the 15-gene signature classifier in the GSE161158 validation dataset. **B,** KM analysis of RFS according to the 15-gene signature classifier in the GSE26906 validation dataset. **C,** ROC graph showing the sensitivity, specificity, and AUC value of the 15-gene signature to predict relapse in the GSE161158 validation dataset. The red dot indicates the recurrence score threshold that was used to determine sensitivity and specificity. **D,** ROC graph showing the sensitivity, specificity, and AUC value of the 15-gene signature to predict relapse in the GSE26906 validation dataset. The red dot indicates the recurrence score threshold that was used to determine sensitivity and specificity. **E,** Risk of recurrence as a continuous function of the 15-gene signature recurrence score within 10 years after surgery in the GSE161158 validation dataset. The shaded area around the curve represents the lower and upper 95% CI. **F,** Risk of recurrence as a continuous function of the 15-gene signature recurrence score within 10 years after surgery in the GSE26906 validation dataset. The shaded area around the curve represents the lower and upper 95% CI.

For validation of the identified 15-gene prognostic signature, we analyzed two additional publicly available stage II colorectal cancer patient cohorts that were not included in the initial six training cohorts. The 15-gene signature was significantly associated with RFS in both publicly available validation cohorts (GSE161158 cohort: HR = 5.81; *P* = 3.57*10^−4^, GSE26906 cohort: HR = 7.698; *P* = 7.26*10^−8^; Fig. 6A and B). The signatures showed a sensitivity of 64.29% and a specificity of 81.67% with an AUC value of 0.796 to predict relapse in the GSE161158 cohort (Fig. 6C) and a sensitivity of 61.54% and a specificity of 78.33% with an AUC value of 0.752 in the GSE26906 cohort (Fig. 6D). Finally, the risk of recurrence at 10 years increased continuously and significantly (GSE161158: *P* = 0.0002; GSE26905: *P* = 0.0003) with increasing recurrence score in both validation cohorts (Fig. 6E and F). Taken together, our results demonstrate that the 15-gene signature represents a robust and consistent biomarker for the prediction of relapse in multiple training and validation stage II colorectal cancer patient cohorts.

Next, we compared the performance of the 15-gene signature with the 7-gene Oncotype DX colon signature, which is the most widely commercially used signature for the prognostication of stage II colorectal cancer. The Oncotype DX colon recurrence score was calculated by two algorithms as described in Materials and Methods. In both cases, the Oncotype DX signature was less significantly associated with RFS than the 15-gene signature characterized here [our algorithm (see Supplementary Fig. S2A and S2B): HR = 2.698, *P* = 6.3*10^−8^, sensitivity = 62.16%, specificity = 55.5%, AUC = 0.633; Oncotype algorithm (see Supplementary Fig. S2C and S2D): HR = 1.83, *P* = 1.88*10^−4^, sensitivity = 57.43%, specificity = 57.36%, AUC = 0.593]. On the basis of the 15-gene signature, 187 patients (23.6%) were designated as high-risk patients, whereas the Oncotype DX colon signature determined 214 high-risk patients (27%) among the 792 patients with colorectal cancer of the pooled training cohort. Therefore, 27 patients would be spared from receiving chemotherapy based on the 15-gene signature described here when compared with the 7-gene Oncotype DX colon signature. The 15-gene signature also showed a better performance than the Oncotype DX colon signature in all six training cohorts and the two publicly available validation cohorts (Supplementary Table S3). In the later, the accuracy in the GSE26906 cohort was 73.26% and 53.49% for the 15-gene and the Oncotype signatures, respectively. The accuracy in the GSE161158 cohort was 78.38% and 62.16% for the 15-gene and the Oncotype signatures, respectively. The concordance of the 15-gene and the Oncotype signatures was also compared patient by patient (Supplementary Table S4).

Finally, we compared the 15-gene signature with the current American Society of Clinical Oncology (ASCO) guideline for patients with stage II colorectal cancer (7). From the risk factors that are described in the ASCO guideline, the pT information was available for the TCGA, GSE39582, GSE33113, and the in-house cohorts. We determined the number of high-/low-risk patients for each of these cohorts based on the pT status (high risk: pT4, low risk: pT3) or the 15-gene signature (Supplementary Table S5). Generally, the number of high-risk patients identified on the basis of the 15-gene signature was higher than the number of high-risk patients identified on the basis of the pT status. However, the ASCO guideline also suggests the identification of high-risk patients based on other risk factors, including sampling of fewer than 12 lymph nodes in the surgical specimen, perineural or lymphovascular invasion, intestinal obstruction, and tumor perforation. However, the information about these factors was not available for the publicly available cohorts and not collected for the in-house cohort and cannot be retrieved anymore due to dual anonymization of the samples.

## Discussion

Currently, there is no consensus on the postsurgery treatment options for patients with stage II colorectal cancer (2). The advancement of methods for improved stratification of patients with low versus high risk of relapse would allow to identify patients who will benefit from chemotherapy, while those who have a high chance of cure by surgery alone could avoid toxic chemotherapy that is unlikely to provide survival benefits for them. Here we identified and validated a 15-gene expression signature that robustly predicts the risk for relapse in patients with stage II colorectal cancer in multiple training and validation cohorts. Importantly, the signature was an independent and most significant marker for the prediction of RFS and OS in a multivariate analysis that included pT, grade, and MSI status. However, the combination of pT and the 15-gene score was superior to the 15-gene score alone in predicting the patient outcome.

Single-gene/protein biomarkers usually only classify patients into low- or high-risk groups. However, our 15-gene signature provides a continuous recurrence score with a range from 0 to 15. Because the risk of recurrence increased continuously and significantly with increasing recurrence score in training and validation cohorts, the signature allows a better classification of patients according to their risk of relapse than a binary classification. This is most relevant for patients with intermediate risk scores, for which additional clinicopathologic risk factors, such as age or general health condition might be considered for adjuvant treatment decision. This would not be possible with binary patient classification. Therefore, the 15-gene signature combined with clinicopathologic risk factors could be used for prognostication and risk stratification of patients with stage II colorectal cancer. Incorporation of the signature into the clinical context may allow better informed decisions on adjuvant therapy for patients with stage II colorectal cancer.

We analyzed the mRNA expression in the in-house cohort using NanoString technology. This approach was chosen because the tumor material was stored as FFPE material for a long period (up to 13 years), which leads to RNA degradation and consequently a decreased qPCR performance (17). NanoString technology does not require nucleic acid amplification by PCR and is therefore more suitable to analyze FFPE tissues with high RNA degradation. It has been demonstrated that by using NanoString, mRNA quantification was similarly efficient, whether RNA was isolated from FFPE or fresh tissue (18, 19). However, when using fresh tissue or FFPE material that has not been stored for long time, also qPCR assays could be used to analyze the 15-gene signature. This would exclude the requirement for the expensive NanoString instrumentation and would make the approach more cost effective.

Several genes from the 15-gene signature play important roles in cancer. For example, suppression of the junction plakoglobin (JUP) resulted in the repression of colorectal cancer cell migration and invasion (20). Furthermore, high levels of the CD40 ligand (CD40LG) have been associated with poor prognosis in colorectal cancer (21). Moreover, high expression of the potassium voltage-gated channel subfamily D member 2 (KCND2) was associated with poor survival in gastric cancer (22). Interestingly, the Atonal bHLH transcription factor 1 (ATOH1), which was associated with good survival in our analyses of patients with stage II colorectal cancer, has been also associated with good survival in colorectal cancer, independent of stage (23). *ATOH1* is frequently deleted or methylated in colorectal cancer and plays a tumor suppressive role by inducing the expression of p21 and suppression the expression of Cyclin D1 (24, 25).

The 247 unfavorable genes among the initially identified 394 prognostic genes showed the highest enrichment within the EMT MSigDB gene set. Some of these genes associated with poor prognosis also encode potential therapeutic targets for colorectal cancer treatment. Besides *PGK1*, which we described in the results part, another unfavorable prognostic gene *MMP14* conferred chemoresistance in the *APC*Min-mouse model of intestinal cancer (26). Moreover, antibody-mediated blockade of MMP14 decreased tumor progression in mammary tumor mouse models (27). We compared the performance of the 15-gene signature with the 7-gene Oncotype DX colon signature, which is the most widely used signature for the prognostication of stage II colorectal cancer. In a pooled dataset consisting of 792 patients from six publicly available stage II colorectal cancer patient cohorts, our 15-gene signature showed a better performance compared with the 7-gene Oncotype DX colon signature, with respect to significance, HR, AUC, sensitivity, and specificity. However, the Oncotype DX colon signature has been tested in thousands of patients. Therefore, it is premature to make the conclusion which signature can better determine patients who will be the most appropriate for chemotherapy. The Oncotype signature score was originally calculated on the basis of qPCR expression data. However, in publicly available cohorts the expression was analyzed by microarray or RNA-seq assays. Therefore, the calculation of the Oncotype score from these results might deviate from the qPCR-based score.

Taken together, by analyzing multiple stage II colorectal cancer patient training cohorts, we identified a 15-gene expression signature, which was validated in multiple, independent colorectal cancer cohorts. Therefore, the 15-gene signature represents a robust prognostic biomarker for stage II colorectal cancer. Yet, the 15-gene signature should be analyzed in additional cohorts to further solidify its prognostic value. Furthermore, combined analysis of the signature and clinico-pathologic parameters might further improve the prognostic sensitivity and specificity of the signature. Finally, to determine the predictive value of the signature with respect to chemotherapy benefit, the signature should be analyzed in a randomized and controlled study, such as the Quick and Simple and Reliable (QUASAR) study (28). Furthermore, the predictive value of the 15-gene signature to determine the benefit of chemotherapy should be analyzed in a prospective study, the gold standard for the validation of predictive markers.

## Supplementary Material

Supplementary Table S1 Supplementary Table S1 shows a list of 394 genes that were significantly associated with survival in CRC patient cohorts.

Supplementary Table S2 Supplementary Table S2 shows a subset of 61 genes that were highly significantly associated with survival in CRC patient cohorts.

Supplementary Table S3 Supplementary Table S3 shows a summary and performance of prognostic signatures in CRC patient cohorts.

Supplementary Table S4 Supplementary Table S4 shows the concordance of the 15-gene and the Oncotype DX colon signatures.

Supplementary Table S5 Supplementary Table S5 shows the concordance of the 15-gene and the Oncotype DX colon signatures.

Supplementary Figure S1 Supplementary Figure S1 shows pathway analysis and clinico-pathological associations of unfavorable prognostic genes.

Supplementary Figure S2 Supplementary Figure S2 shows the association of the Oncotype DX colon signature with relapse free survival in the pooled stage II cohort.
