# Supplementary material for: Development and Validation of a 15-gene Expression Signature with Superior Prognostic Ability in Stage II Colorectal Cancer
Source: Cancer Res Commun. 2023 Aug 30;3(8):1689–700. doi: 10.1158/2767-9764.CRC-22-0489 (PMC10467603; doi:10.1158/2767-9764.CRC-22-0489)
Supplement: Supplementary Table S3 — shows a summary and performance of prognostic signatures in CRC patient cohorts. [file crc-22-0489-s03.docx]

**Table S3**. Summary of prognostic performance of indicated signatures in training and validation cohorts

| Signature | Cohort type | Cohort | AUC | Sensitivity | Specificity | Accuracy |
| --- | --- | --- | --- | --- | --- | --- |
| 61-gene  signature | Training cohorts | TCGA stage II | 0.856 | 85.00 | 73.37 | 75.59 |
|  |  | GSE39582 stage II | 0.939 | 86.21 | 90.36 | 89.41 |
|  |  | GSE14333 stage II | 0.979 | 100 | 87.5 | 89.36 |
|  |  | GSE33113 stage II | 0.986 | 100 | 90.14 | 92.22 |
|  |  | GSE17538 stage II | 0.949 | 100 | 90.48 | 91.67 |
|  |  | GSE37892 stage II | 0.989 | 100 | 93.85 | 94.52 |
|  |  | pooled stage II | 0.937 | 89.29 | 89.61 | 89.53 |
|  | Validation cohorts | in house cohort | 0.613 | 50 | 72.54 | 65.69 |
|  |  | GSE26906 stage II | 0.7878 | 69.23 | 73.33 | 72.09 |
|  |  | GSE161158 stage II | 0.8768 | 78.58 | 83.33 | 82.43 |
| 15-gene  signature | Training cohorts | TCGA stage II | 0.771 | 70 | 78.11 | 76.56 |
|  |  | GSE39582 stage II | 0.788 | 72.41 | 65.99 | 67.45 |
|  |  | GSE14333 stage II | 0.9129 | 92.86 | 77.5 | 79.79 |
|  |  | GSE33113 stage II | 0.937 | 94.74 | 76.06 | 80.00 |
|  |  | GSE17538 stage II | 0.951 | 100 | 82.54 | 84.72 |
|  |  | GSE37892 stage II | 0.885 | 87.5 | 87.69 | 87.67 |
|  |  | pooled stage II | 0.784 | 75 | 67.44 | 68.85 |
|  | Validation cohorts | in house cohort | 0.812 | 90.32 | 80.99 | 83.82 |
|  |  | GSE26906 stage II | 0.752 | 61.54 | 78.33 | 73.26 |
|  |  | GSE161158 stage II | 0.796 | 64.29 | 81.67 | 78.38 |
| Oncotype DX  colon signature, our algorithm | Training cohorts | TCGA stage II | 0.571 | 50 | 64.5 | 61.72 |
|  |  | GSE39582 stage II | 0.591 | 72.41 | 39.09 | 46.67 |
|  |  | GSE14333 stage II | 0.733 | 64.29 | 72.5 | 71.28 |
|  |  | GSE33113 stage II | 0.502 | 57.89 | 40.85 | 44.44 |
|  |  | GSE17538 stage II | 0.624 | 44.44 | 82.54 | 77.78 |
|  |  | GSE37892 stage II | 0.779 | 87.5 | 63.08 | 65.75 |
|  |  | pooled stage II | 0.633 | 62.16 | 55.5 | 56.75 |
|  | Validation cohorts | GSE26906 stage II | 0.596 | 42.31 | 68.33 | 60.47 |
|  |  | GSE161158 stage II | 0.763 | 71.43 | 63.33 | 64.87 |
| Oncotype DX colon signature, Oncotype algorithm | Training cohorts | TCGA stage II | 0.517 | 52.5 | 52.07 | 52.15 |
|  |  | GSE39582 stage II | 0.589 | 56.9 | 56.35 | 56.47 |
|  |  | GSE14333 stage II | 0.639 | 57.1 | 67.5 | 65.96 |
|  |  | GSE33113 stage II | 0.515 | 52.63 | 53.52 | 52.89 |
|  |  | GSE17538 stage II | 0.748 | 66.67 | 92.06 | 88.89 |
|  |  | GSE37892 stage II | 0.641 | 75 | 53.85 | 56.16 |
|  |  | pooled stage II | 0.593 | 57.43 | 57.36 | 57.88 |
|  | Validation cohorts | GSE26906 stage II | 0.5724 | 57.69 | 51.67 | 53.49 |
|  |  | GSE161158 stage II | 0.6583 | 64.29 | 61.67 | 62.16 |
