# Supplementary material for: Development and Validation of a 15-gene Expression Signature with Superior Prognostic Ability in Stage II Colorectal Cancer
Source: Cancer Res Commun. 2023 Aug 30;3(8):1689–700. doi: 10.1158/2767-9764.CRC-22-0489 (PMC10467603; doi:10.1158/2767-9764.CRC-22-0489)
Supplement: Supplementary Table S5 — shows the concordance of the 15-gene and the Oncotype DX colon signatures. [file crc-22-0489-s05.docx]

**Table S5.** Comparison of the number of high/low risk patients based on the ASCO stage2 CRC guideline and the 15-gene signature

|  | ASCO guideline (pT) | | 15-gene signature | |
| --- | --- | --- | --- | --- |
| cohort | low risk  (pT3) | high risk  (pT4) | low risk  (low score) | high risk  (high score) |
| in house stage2 | 164 (80.39) | 40 (19.61) | 120 (59.11) | 83 (40.89) |
| TCGA stage2 | 216 (93.51) | 15 (6.49) | 149 (71.63) | 59 (28.37) |
| GSE39582 stage2 | 196 (79.03) | 52 (20.97) | 221 (86.67) | 34 (13.33) |
| GSE33113 stage2 | 83 (92.22) | 7 (7.78) | 78 (86.67) | 12 (13.33) |
