# Supplementary material for: Development and Validation of a 15-gene Expression Signature with Superior Prognostic Ability in Stage II Colorectal Cancer
Source: Cancer Res Commun. 2023 Aug 30;3(8):1689–700. doi: 10.1158/2767-9764.CRC-22-0489 (PMC10467603; doi:10.1158/2767-9764.CRC-22-0489)
Supplement: Supplementary Figure S1 — shows pathway analysis and clinico-pathological associations of unfavorable prognostic genes. [file crc-22-0489-s06.docx]

**Figure S1. Pathway and single gene analysis of unfavorable prognostic genes**

A and B. Gene set enrichment analysis of the 247 unfavorable genes. The 10 MSigDB Hallmark (A) and KEGG (B) gene sets with the most significant enrichment are shown

C. Forest plot showing HRs for relapse-free survival by comparing patients with high versus low expression of *PGK1* in indicated cohorts. Dots represent HRs and horizontal lines show 95% confidence intervals. P values were calculated using the log-rank method.

D. PGK1 dependency score in CRC cell lines. Data was derived from the Cancer Dependency Map project CRISPR and RNAi datasets.

E. Forest plot showing fold changes in *PGK1* expression between colorectal cancers and matched adjacent normal colonic mucosa for indicated cohorts. Dots represent fold change and horizontal lines show 95% confidence intervals. P values were calculated using paired t test.
