# Supplementary material for: Development and Validation of a 15-gene Expression Signature with Superior Prognostic Ability in Stage II Colorectal Cancer
Source: Cancer Res Commun. 2023 Aug 30;3(8):1689–700. doi: 10.1158/2767-9764.CRC-22-0489 (PMC10467603; doi:10.1158/2767-9764.CRC-22-0489)
Supplement: Supplementary Figure S2 — shows the association of the Oncotype DX colon signature with relapse free survival in the pooled stage II cohort. [file crc-22-0489-s07.docx]

**Figure S2. Comparison with the Oncotype DX colon 7-gene signature**

A. Kaplan–Meier analysis of RFS according to the Oncotype DX colon signature classifier in the pooled training dataset. The signature recurrence score was calculated according to our algorithm based on 0/1 scoring as described in Methods.

B. ROC graph showing the sensitivity, specificity and AUC value of the Oncotype DX colon signature to predict relapse in the pooled training dataset. The signature recurrence score was calculated according to our algorithm based on 0/1 scoring as described in Methods. The red dot indicates the recurrence score threshold that was used to determine sensitivity and specificity.

C. Kaplan–Meier analysis of RFS according to the Oncotype DX colon signature classifier in the pooled training dataset. The signature recurrence score was calculated by using the Oncotype DX algorithm as described in Methods.

D. ROC graph showing the sensitivity, specificity and AUC value of the Oncotype DX colon signature to predict relapse in the pooled training dataset. The signature recurrence score was calculated by using the Oncotype DX algorithm as described in Methods. The red dot indicates the recurrence score threshold that was used to determine sensitivity and specificity.
